# Enablers and barriers to post-discharge follow-up among women who have undergone a caesarean section: experiences from a prospective cohort in rural Rwanda

**DOI:** 10.1186/s12913-022-08137-5

**Published:** 2022-06-02

**Authors:** Theoneste Nkurunziza, Robert Riviello, Frederick Kateera, Edison Nihiwacu, Jonathan Nkurunziza, Magdalena Gruendl, Stefanie J. Klug, Bethany Hedt-Gauthier

**Affiliations:** 1 Partners In Health/Inshuti Mu Buzima, KG 9 Avenue 46, PO Box 3432, Kigali, Rwanda; 2 Epidemiology Department of Sports and Health Sciences, Technical University of Munich, Munich, Germany; 3 Department of Global Health and Social Medicine, Harvard Medical School, Boston, USA; 4 Center for Surgery and Public Health, Brigham and Women’s Hospital, Boston, USA

**Keywords:** Post-discharge care, Global surgery, Sub-Saharan Africa, mHealth, Surgical site infections, Follow-up

## Abstract

**Background:**

Caesarean sections account for roughly one third of all surgical procedures performed in low-income countries. Due to lack of standardised post-discharge follow-up protocols and practices, most of available data are extracted from clinical charts during hospitalization and are thus sub-optimal for answering post-discharge outcomes questions. This study aims to determine enablers and barriers to returning to the hospital after discharge among women who have undergone a c-section at a rural district hospital in Rwanda.

**Methods:**

Women aged ≥ 18 years who underwent c-section at Kirehe District Hospital in rural Rwanda in the period March to October 2017 were prospectively followed. A structured questionnaire was administered to participants and clinical data were extracted from medical files between March and October 2017. At discharge, consenting women were given an appointment to return for follow-up on postoperative day 10 (POD 10) (± 3 days) and provided a voucher to cover transport and compensation for participation to be redeemed on their return. Study participants received a reminder call on the eve of their scheduled appointment. We used a backward stepwise logistic regression, at an α = 0.05 significance level, to identify enablers and barriers associated with post-discharge follow-up return.

**Results:**

Of 586 study participants, the majority (62.6%) were between 21–30 years old and 86.4% had a phone contact number. Of those eligible, 90.4% returned for follow-up. The predictors of return were counselling by a female data collector (OR = 9.85, 95%CI:1.43–37.59) and receiving a reminder call (OR = 16.47, 95%CI:7.07–38.38). Having no insurance reduced the odds of returning to follow-up (OR = 0.03, 95%CI:0.03–0.23), and those who spent more than 10.6 Euro for transport to and from the hospital were less likely to return to follow-up (OR = 0.14, 95%CI:0.04- 0.50).

**Conclusion:**

mHealh interventions using calls or notifications can increase the post-discharge follow-up uptake. The reminder calls to patients and discharge counselling by a gender-matching provider had a positive effect on return to care. Further interventions are needed targeting the uninsured and patients facing transportation hardship. Additionally, association between counselling of women patients by a female data collector and greater return to follow-up needs further exploration to optimize counselling procedures.

**Supplementary information:**

The online version contains supplementary material available at 10.1186/s12913-022-08137-5.

## Introduction

Caesarean sections (c-sections) are the most commonly performed major surgical procedure globally and account for roughly a third of all surgical procedures performed in low-income countries (LICs)[1]. Over the past two decades, there has been an increase in c-section rates from 6.7% to 19.1% globally, with a more modest increase (6.0%) observed in LICs [2]. However, increased access to c-sections in sub-Saharan Africa (SSA) has been linked to reductions in maternal and neonatal mortality rates [3–5].

For optimal maternal and child outcomes, the World Health Organization (WHO) recommends general postpartum follow-up on day 3 (48–72 h), between days 7–14, and at six weeks after delivery [6]. This could lead to early detection and treatment of complications [7]. However, in LICs, some patients face geographical and financial barriers as they strive to return for follow-up care [8]. In a systematic review on factors affecting postpartum follow-up, dependency of women on their husbands, lack of information, absence of complications, unsatisfactory customer care, husband’s education and occupation, and large family size and household income were identified as impeding the decision to return to care [9].

Particular to women who undergo c-section, surgical site infections (SSI) are very common after their discharge [10, 11]. Reasons behind this can be poor living conditions, poor sanitation, patient’s poor information, delay in seeking or accessing healthcare, poor quality of wound care just to name a few [12, 13]. SSIs introduce a higher morbidity, mortality, and social economic burden on patients and health systems [14–16]. The SSI prevalence and related burden is higher in low-resourced settings [17], such as SSA countries, that report rates of post-caesarean SSI as high as 41.9% [18]. Due to the lack of active surveillance and standard follow-up after a patient is discharged from the hospital, these SSI rates are likely underestimated [19] since 60% of SSIs develop after a patient discharge [20].

In Rwanda, the caesarean section delivery rate in is 14.9% [21]. The community-based health insurance (CBHI) is implemented nationwide to facilitate access to healthcare because majority (55%) of the population is living in poverty [22]. The yearly premiums for this scheme range from 3.2 Euro to 7.6 Euro per head referring to the Ubudehe category. This is the 4-level socio-economic classification system of the population based on household welfare status, whereby Ubudehe category 1 are the poorest and category 4 the wealthiest [23]. Category 1 patients have their entire medical bills subsidized by the government.

Moreover, in addition to implementing WHO postnatal care guidelines [24], general maternal care initiatives were put in place. For example, the Ending Preventable Maternal Mortality initiative aimed to decrease maternal mortality to 70 per 100,000 live births by 2030 [25, 26], and community health workers (CHWs)’ programme. The network of CHWs plays a critical role in maternal and neonatal care during the prenatal and postnatal period in their local communities[27]. However, these CHWs are not trained to support surgical follow-up [28, 29].

In spite of those efforts implemented to increase access to healthcare and improve maternal and child outcomes, the 2015 Rwandan National Institutes of Statistics (NISR) report shows that the same socio-economic and geographical barriers to post-partum follow-up care faced by other LICs are prevalent in Rwanda [30]. This results into only 43% of women receiving at least the first post-partum visit [18] and increases the odds of developing SSI and delaying its diagnosis and treatment [31]. To the best of our knowledge, little was done in regards to the particular and additional needs of caesarean patients prone to SSI after their discharge. Further, little is known about their post-discharge follow-up, particularly in rural areas where patients face long distances, difficult-to-access terrains, and financial restrictions to access healthcare. As part of our prospective cohort study among women who undergo a c-section in rural Rwanda, we invited women and supported them with transportation vouchers and reminder calls to return for a study-specific follow-up visit at postoperative day (POD) 10 (± 3 days) at a rural district hospital in Rwanda. Here, we report the enablers and barriers for returning for follow-up to inform strategies for effective postoperative care seeking in this context.

## Methods

### Study setting

This prospective study included women who underwent c-section between March and October 2017 at Kirehe District Hospital (KDH) in rural Rwanda. KDH is managed by the Rwandan Ministry of Health (RMoH) and receives technical and financial support from Partners In Health/Inshuti Mu Buzima (PIH/IMB), a US-based, non-governmental health organization. At KDH, c-sections are mainly performed by general practitioners (GP) and are the most prevalent surgical intervention. KDH has a full-time obstetrician gynecologist on its staff under PIH/IMB support who provides mentorship to available GPs and manages the most complex obstetric and gynecologic cases.

In the Rwandan health care system, patients present first to the nearest health center for primary health care, basic evaluation, and treatment by nurses. These nurses transfer any cases requiring management by a GP to the district hospitals. District hospitals in Rwanda provide a secondary level of care, including minor and some major surgical interventions, as well as management of surgical emergencies, such as c-sections. These hospitals are staffed with GPs, nurses, midwives, other paramedical health practitioners, and administrative personnel [32]. District hospitals transfer complex cases in need of management by a specialist to tertiary care facilities, mostly in the capital city, Kigali [33].

A woman who undergoes a c-section is admitted to the postpartum ward for monitoring and postoperative care and, if she does not experience complications, is routinely discharged on POD 3. The decision to discharge the patient is made by a GP who completes a discharge form with notes on the patient’s in-hospital management and the plan for post-discharge follow-up. A ward midwife or nurse then counsels the patient and gives discharge instructions, including operative wound care, a follow-up date for her wound dressing change at her nearest health center, details about any post-discharge medications, and neonatal care.

### Study design and population

Data used for this study are a subset of data collected in the larger prospective cohort study where consenting adult women (≥ 18 years of age) who underwent c-section at KDH between March 22^nd^ and October 18^th^, 2017 were enrolled and followed up for SSI detection [34]. This window was selected based on funding availability and implementation logistics but did give us the advantage of covering both the rainy and dry seasons. Patients from Mahama Refugee Camp were excluded given that their return would depend on the camp management. Patients from outside the KDH catchment area were also excluded given that they were likely to follow-up at their nearest health facility. Further, patients who were still hospitalized by POD 7 or readmitted before their follow-up date were also excluded.

### Implementation of the study

At discharge, enrolled women received discharge counselling detailing the follow-up plan by study trained data collector. Each study participant was given a return date to the study-specific SSI screening clinic at KDH on POD 10 (± 3 days). To prevent transport costs from being a financial barrier to study participants returning to KDH, we provided a voucher on discharge that would be redeemed upon return to cover the transport costs and compensate the women’s time participating in the study. To determine transportation fees, we used the guide set by PIH/IMB in Kirehe based on distance from each district zones to the hospital.

The study clinics were held on Tuesdays and Thursdays. Study participants were called on the eve of their scheduled appointment to remind them of their clinic schedule. When a patient missed her first clinic appointment, she was called again and was given a second appointment on the following study clinic day. At the study clinic, data collectors recorded their attendance status and a GP implemented the SSI screening protocol (results of the SSI screening is reported elsewhere) [31, 34].

### Data collection

The study employed five trained data collectors, one female and four males, with clinical backgrounds. The questionnaire that had been developed and tested in Haiti [35] was translated in Kinyarwanda by the local study team and tested on 12 individuals for comprehension in March 2017. It was revised accordingly before the start of the study. The data collectors identified patients who had undergone c-section from the operating room registry and located them in the maternity postoperative room on POD 1. They explained the study aims, benefits and risks of participating in the study to eligible participants, and invited them to voluntarily consent to participate in the study. Those who consented were enrolled as study participants. The data collectors administered a structured questionnaire collecting demographic data from enrolled study participants. Clinical data were extracted from medical files. Study participants’ access to a phone was also documented. A patient was confirmed to have access to a phone when she owned one, had a phone available in her household, or she gave the number of a neighbor where she could be reached.

A patient was considered as having comorbidity when she had any of the following underlying disease conditions prior to c-section: HIV/AIDS, diabetes, hypertension and other cardio-vascular disorders. Post-operative complications included any morbidity that occurred after surgery and prior to discharge as assessed by a GP or an obstetrician-gynaecologist and documented in the medical file. The documented complications were haemorrhage, fever, organ disfunction such as respiratory depression or urinary dysfunction, wound dehiscence, return to operating room, and any other post-operative abnormality diagnosed post-operatively during the hospital stay. The total length of hospital stay and post-operative length of stay were calculated by subtracting the date of discharge from the date of admission and date of surgery, respectively.

In addition to demographic and clinical data, we obtained rainfall data corresponding to the study clinic days from the Rwandan Metrological Agency. This agency uses satellite and has six stations in Kirehe. Each woman had two rainfall data points attached to her study visit. First, we used the data corresponding to the station closest to the participant’s residence on the day of her study follow-up visit. Second, we also collected the rainfall data for the station closest to KDH for the day she was attending the study clinic. These data were used to analyze whether the rain had negative effect on participants’ return on their appointments. All data were entered into REDCap, a secure web application that can support both online or offline data collection [36].

### Analysis and statistics

The primary outcome for this study was the return to the study follow-up visit, defined as coming to the first or the second study clinic appointment. We used descriptive statistics to report study participants demographic and clinical characteristics. We converted Rwandan francs (FRW) to Euro using the 942 FRW/Euro rate, referring to the then average central bank exchange rate [37]. To identify predictors of return to follow-up care, we performed univariable logistic regression to determine variables eligible for a multivariable logistic regression model (Supplementary table S1). Variables significant at an α = 0.1 significance level were considered for the reduced model. We built the model using backward stepwise selection, stopping when the remaining covariates were significant at α = 0.05 significance level. We report the odds ratios (ORs), 95% confidence intervals (95%CIs) and p-values from the multivariable analysis. All analyses were performed using Stata v15 (College Station, TX: StataCorp LP).

## Results

Of 746 women who underwent c-section, 586 (78.6%) were eligible for study enrollment. The majority (62.6%, *n* = 338) were between 21–30 years, married (43.0%, *n* = 252), with primary education (70.0%, *n* = 410), and were insured with CBHI (95.1%, *n* = 557). Most of the women were farmers (86.7%, *n* = 508), earned less than 31.8 Euro/month (92.7%, *n* = 543), and had access to a phone (86.4%, *n* = 506). Of those with recorded information on transport vouchers, 392 (66.9%) were issued between 5.3 and 10.6 Euro (Table 1). Fetal distress was the leading indication of c-Sect. (32.2%, *n* = 189) followed by previous scar (29.5%, *n* = 173). The majority of patients (73.2%, *n* = 423) were discharged by POD 3. The male data collectors enrolled and consented a majority of the study participants (84.1%, *n* = 493). Forty-one (7.0%) of study participants were exposed to rain, either at home or KDH, on their first appointment date or, for those who did not attend their first appointment date, on their second appointment date (Table 2).

**Table 1:** Demographic characteristics of study participants (*N* = 586)

**Variables**	**Frequency**	**Percent**	
**Age**			
20 years and younger	73	12.5	
21–30 years old	367	62.6	
31–39 years old	128	21.8	
40 years and older	18	3.1	
**Marital status**			
Single	209	35.7	
Married	252	43.0	
Living with a partner	119	20.3	
Separated (divorced or widowed)	6	1.0	
**Education level**			
No education	47	8.0	
Primary education	410	70.0	
Secondary education or higher	129	22.0	
**Occupation**			
Farmer	508	86.7	
Employed, trader	43	7.3	
Housewives	35	6.0	
**Type of insurance**			
No insurance	6	1.0	
Community-Based Health Insurance (CBHI)	557	95.1	
Private insurance	23	3.9	
**Monthly household income†**			
Less than < 31.8 Euro/month	543	92.7	
31.8 Euro and above	43	7.3	
**Does the patient have phone contact?**			
No	71	12.1	
Yes	506	86.4	
Missing	9	1.5	
**Amount of transportation voucher fees**			
Up to 5.30 Euro†	119		20.3
> 5.30–10.60 Euro	392		66.9
Greater than 10.60 Euro	62		10.6
Missing	13		2.2

^†^ = converted from FRW using the 942 FRW/Euro rate referring to the then average central bank exchange rate

**Table 2:** Clinical characteristics of study participants (*n* = 586)

Variables	Frequency	Percent
**Co-morbidity**^**&**^		
No	571	97.4
Yes	15	2.6
**An**a**esthesia type**		
General	14	2.4
Loco-regional	572	97.6
Indication to C-section*		
Foetal distress	189	32.3
Previous scar	173	29.5
Prolonged labour	68	11.6
Malpresentation	80	13.7
Obstructed labour	70	12.0
Cord and membrane dystocia	45	7.7
Hypertensive disorders	7	1.2
Uterine pre/rupture	8	1.4
Hypotonic dysfunction	21	3.6
Other indications	13	2.2
Post-operative complications**		
No	574	98.0
Yes	12	2.0
**Total length of stay (LOS)**		
Within 3 days	338	57.7
More than 3 days	248	42.3
**Post-operative LOS**		
Within 3 days	423	73.2
4–7 days	163	27.8
**Duration of post-surgery antibiotic therapy**		
No post-operative antibiotic	21	3.6
1–3 days	442	75.4
More than 3 days	123	21.0
**Counselling data collector**		
Male data collectors	493	84.1
Female data collector	86	14.7
Missing	7	1.2
**Was it raining on the patient's appointment day?**		
No	545	93.0
Yes	41	7.0

^&^ Comorbidities included HIV/AIDS, diabetes, hypertension and other cardio-vascular disorders

^*^ = Percent total greater that 100% due to possibility of more than 1 indication for a C-section decision

^**^ = Post-operative complications included haemorrhage, fever, organ disfunction such as respiratory depression or urinary dysfunction, wound dehiscence, return to operating room, and any other post-operative abnormality diagnosed post-operatively during the hospital stay

**Fig. 1:**
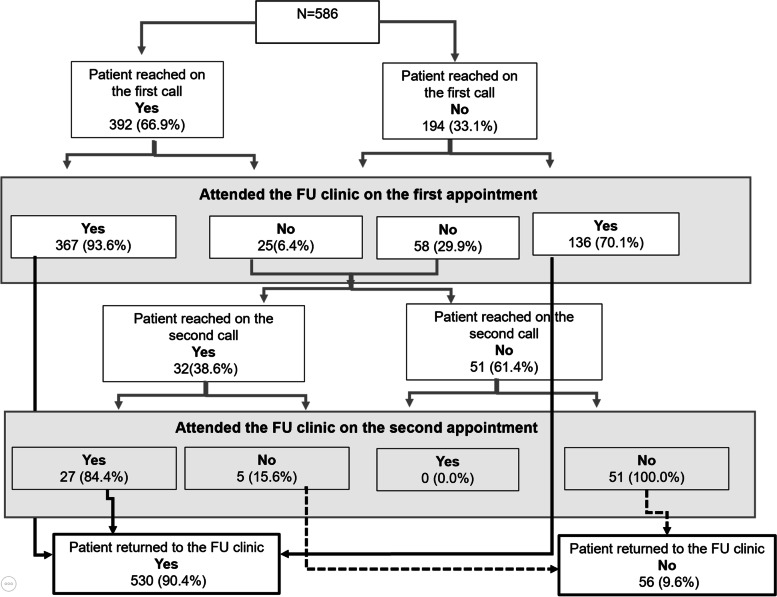
Flowchart of the study implementation and follow-up rates. Dotted lines represent women who did not return to follow-up clinic. Full lines display those who returned to care

Nearly all participants returned to the study follow-up clinic (90.4%, *n* = 530), most (85.8%, *n* = 503) on the date of their first clinic appointment. The phone call reached 72.3% (*n* = 424) of participants to remind them of either of their appointments. Of those reminded, 93.6% (*n* = 367) attended their first appointment. Of 194 participants who were not reached by the first reminder phone call, 136 (70.1%) returned to the clinic, all on the first appointment. Of 83 who did not show up at the first visit, 32 (38.6%) were reached by the second reminder call, and 27 of them (84.4%) attended their second appointment. Overall, 56 participants (9.6%) were not reached at all on the phone call and did not attend the clinic (Fig. 1).

In the reduced model of the multivariable analysis, having a female data collector enrolling and counselling study participants was associated with higher return to care compared to male counterparts (OR = 9.85, 95%CI:1.43–37.59). Study participants who were called and reminded of their appointment had 16-times greater odds of returning to their follow-up clinic (95%CI: 7.07–38.38). Study participants who had no insurance were 97% less likely to return to their follow-up clinic as compared to those who had CBHI (OR:0.03, 95%CI: 0.03- 0.23). Study participants who were expected to spend more than 10.6 Euro for a round-trip ticket to return to the follow-up clinic were 86% less likely to return (OR = 0.14, 95%CI:0.04–0.50) (Table 3). Marital status, education level, type of insurance, and post-operative length of stay were included in the regression model but did not remain significant until the final model.

**Table 3:** Enablers and barriers of the return to a follow-up clinic after c-section (multivariable regression model) *n* = 567

	**FULL MODEL (*n* = 567)**			**REDUCED MODEL (*n* = 567)**		
	**OR**	**95% CI**	***P***	**OR**	**95% CI**	***P***
**Marital status**						
Married	1					
Single	0.71	(0.31–1.62)	0.415			
Living with a partner	0.62	(0.25–1.57)	0.314			
**Education level**						
Primary education	1					
No education	0.34	(0.12–0.99)	0.048			
Secondary education or higher	1.72	(0.45–6.63)	0.429			
**Type of insurance**						
CBHI	1					
No insurance	0.034	(0.03–0.33)	0.004	0.03	(0.03–0.23)	0.001
Private insurance	0.67	0.65–6.82)	0.733	0.73	(0.07–7.23)	0.789
**Amount of transportation voucher fees**						
up to 5.3 Euro	1					
> 5.3–10.6 Euro	0.38	(0.13–1.11)	0.078	0.36	(0.13- 1.02)	0.055
Greater than 10.6 Euro	0.13	(0.03–0.52)	0.004	0.14	(0.04- 0.50)	0.003
**Post-operative length of stay**						
Within 3 days	1					
4- 7 days	0.57	(0.27–1.19)	0.135			
**Counseling data collector**						
Male data collectors	1					
Female data collector	9.65	(1.22–45.95)	0.031	9.85	(1.43–37.59)	0.020
**Was the patient reminded of her appointment?**						
No	1					
Yes	17.30	(7.31–40.92)	< 0.001	16.47	(7.07- 38.38)	< 0.001
**Was it raining on the patient's appointment day?**						
No	1					
Yes	1.47	(0.40–5.41)	0.564			

## Discussion

In this study, we identified several factors associated with the participants’ return to the study follow-up visit. Counselling before hospital discharge and a reminder call on the eve of the follow-up appointment were associated with higher return to follow-up. In contrast, higher costs of transportation and lack of health insurance were associated with reduced likelihood of return for the follow-up visit.

A reminder call on the eve of the follow-up appointment was associated with 16-fold higher chance of return. This reflects the role of mobile health technologies in improving the follow-up of surgical patients after their discharge from the hospital. Phone calls have been shown to be feasible and effective in the post-discharge follow-up and care of obstetric patients in Tanzania [38], and has been shown to optimize follow-up in other non-obstetric settings [39–42]. In this study, more than 80% gave phone contact on which they can be reached, consistent with the reported 71% of households owning cell phones in Rwanda [43]. Our reminder calls reached approximately 70% of study participants. This high phone coverage and the effectiveness of the reminder call should be leveraged in follow-up care activities. Nevertheless, given the burden that reminder calls could present to the already overwhelmed healthcare facilities and providers, less burdensome mHealth tools can be employed. Automated SMS notifications a day before their scheduled clinic could be an alternative with similar effect [44]. This would have an added benefit of reaching those who would otherwise be inaccessible during the call time.

While we attempted to mitigate financial barriers via a transportation voucher, the amount of transportation voucher fees, which served as a proxy of transportation cost from the patient’s home to the hospital, emerged as a significant predictor associated with lower follow-up rates. This implied that the higher the cost of transport, and therefore likely the farther the patient residence, the less likely the patient is to return to follow-up in this rural setting. Further, our study was conducted at a district hospital, the second level of Rwanda healthcare system, which have GPs on its staff; which required participants to travel further distance as compared to distance from home to health centers. Decentralized follow-up of these surgical patients at the nearest health facility may increase follow-up rates; however, as reported in a recent qualitative study [45], health center follow-up is also reported to be both financially and physically burdensome. While our use of vouchers may have offset some of the financial burden, the funding was reimbursed on arrival – a challenge if women could not front the costs – and did not remove physical challenges of travel postpartum and postoperative.

Patients without health insurance had almost no chance to return to the follow-up clinic. Particular to this study clinic, no fees were charged for the service. Yet uninsured patients failed to attend the clinic. For this rural setting, we believe that these were the vulnerable patients particularly from *Ubudehe* category 2 who are not subsidized by the government, and most prone to significantly have lower adherence to health insurance [46]. We attribute their failure to return to impoverishing out-of-pocket incurred at the hospital. Our findings support available data whereby while health insurance increases health service utilization and provides financial protection, that lack of insurance has negative effects on both [47–49]. This suggests that there is a vicious cycle of lack of insurance leading to impoverishment by healthcare costs, and vice-versa, as demonstrated by other studies where poverty was the root cause of uninsured populations in SSA [50]. This group of patients need more attention from healthcare providers to prevent them from being lost-to-follow-up and should benefit from extension of the available social protection programmes.

Discharge counselling by a female data collector was linked to the higher return to care as compared to her male counterparts. Psychological studies suggest that gender-matching improves agreement and emotional bonds that are associated with treatment compliance and retention[51–53]. However, to our knowledge, no study has assessed the gender-matching aspect when it comes to discharge counselling in the context of surgery in SSA. Further studies should explore the rationale behind and benefits of that preference.

Notwithstanding, our cohort has benefited from additional services that could have bettered their return as compared to the current standard of care for national postpartum follow-up. On discharge, study participants received a more detailed counselling by the study data collector regarding their follow-up visit and transport vouchers to facilitate their return. Additionally, they were reminded of their appointment. All these make this cohort unrepresentative of the women who are followed up postoperatively in normal standard of care. Further, according to the Rwandan demographic health survey, only 43% of women benefited from the first visit of standard post-partum care [30]. We suspect that the lost-to-follow-up in post caesarean patients under standard of care may be higher than 10% found in this study and this warrants another study. We believe that our findings would generalize to other women outside of the context of the study if they benefited from the same services since the setting is similar.

Since many changes have taken place after the study was implemented, including the coronavirus pandemic that added burden to healthcare system. We expect that better phone coverage will lead to feasibly reaching more patients by reminder notifications given ongoing efforts to improve access to mobile telephone particularly in rural areas. The current phone ownership in rural area has increased from 54% in 2014 to 67% in 2019 and from 60 to 71% nationally [21, 30].

This study had some limitations to be considered in interpretation. First, the generalization of results is limited given the population that was part of a larger study whose participants received additional services to encourage their return to follow-up clinic. However, the hypotheses generated by this study are relevant to the general population and we suggest further studies to explore the situation and the impact of those add-on services on the return to follow-up care. Second, there was incomplete data on geographical locations and transportation facilities. However, we used the transport fees, following a compensation structure outlined by PIH/IMB at KDH site, as proxy of distance and transport requirements from home to the hospital. Third, there are likely other possible enablers or barriers not considered, such as patient motivation and husband’s influence. Fourth, the male–female data collector ratio was 4:1, which resulted into the female data collector counselling proportionately fewer women. We believe that this hypothesis is worth further exploring. Finally, this study took place in one location in Rwanda; however, the structure of KDH and care protocols in the district are similar to other facilities in Rwanda and the region.

## Conclusions

The study found overall high return to follow-up in this study population; participant reminders via phone calls may contribute to their return for post-discharge follow-up. For those lost-to-follow-up, our findings on the detrimental effects of travel costs support the case for decentralizing follow-up care. Further, patients from rural communities who do not have health insurance represent the population at risk of lost-to-follow-up and so need more support. The association between discharge counselling by the same gender data collector and greater return to follow-up warrants further exploration. While these results are in the context of study-specific follow-up, and may have direct implication for future prospective studies in rural Africa, we believe these lessons learned can inform strategies for effective follow-up care post c-section more broadly.

## Supplementary information

**Additional file 1:** **Table S1.** Univariate logistic regression of predictors of the return to a follow-up clinic after c-section.

## Data Availability

The data that support the findings of this study are available from PIH/IMB but restrictions apply to the availability of these data, which were used under license for the current study, and so are not publicly available. Data are however available from the authors upon reasonable request and with permission of PIH/IMB.

## Abbreviations

CBHI: Community-based health insurance
CHW: Community health worker
C-section: Caesarean sections
GP: General practitioners
KDH: Kirehe District Hospital
LICs: Low-income countries
LOS: Length of stay
NISR: Rwandan National Institutes of Statistics
PIH/IMB: Partners In Health/Inshuti Mu Buzima
POD: Postoperative day
RNEC: Rwanda National Ethics Committee
SSA: Sub-Saharan Africa
SSI: Surgical site infection
WHO: World Health Organization
