# Supplementary material for: Enablers and barriers to post-discharge follow-up among women who have undergone a caesarean section: experiences from a prospective cohort in rural Rwanda
Source: BMC Health Serv Res. 2022 Jun 2;22:733. doi: 10.1186/s12913-022-08137-5 (PMC9160515; doi:10.1186/s12913-022-08137-5)
Supplement: Supplementary file 1 — Additional file 1: Table S1. Univariate logistic regression of predictors of the return to a follow-up clinic after c-section. [file 12913_2022_8137_MOESM1_ESM.docx]

Supplementary table 1: Univariate logistic regression of predictors of the return to a follow-up clinic after c-section (n=586)

|  | **OR** | **95%CI** | **p-value** |
| --- | --- | --- | --- |
| **Age** |  |  |  |
| 21-30 years old | 1 |  |  |
| 20 years and younger | 0.94 | 0.44- 1.20 | 0.868 |
| 31 year and older | 0.73 | 0.39- 1.37 | 0.322 |
| **Marital status** |  |  |  |
| Married | 1 |  |  |
| Single | 0.51 | 0.27- 0.96 | 0.039 |
| Living with a partner | 0.59 | 0.28- 1.26 | 0.172 |
| **Education level** |  |  |  |
| Primary education | 1 |  |  |
| No education | 0.59 | 0.26- 1.34 | 0.203 |
| Secondary education or higher | 3.76 | 1.32- 10.67 | 0.013 |
| **Occupation** |  |  |  |
| Farmer | 1 |  |  |
| Employed, trader | 1.46 | 0.43- 4.88 | 0.543 |
| Housewives | 1.16 | 0.34- 3.94 | 0.807 |
| **Monthly household income** |  |  |  |
| Less than<31.8 Euro/month | 1 |  |  |
| 31.8 Euro and above | 4.74 | 0.64- 35.07 | 0.128 |
| **Type of insurance** |  |  |  |
| CBHI | 1 |  |  |
| No insurance | 0.21 | 0.04- 1.15 | 0.072 |
| Private insurance | 1.08 | 0.25- 4.74 | 0.918 |
| **Does the patient have phone contact?** |  |  |  |
| No | 1 |  |  |
| Yes | 5.59 | 2.91- 10.73 | <0.001 |
| **Amount of transportation voucher fees** |  |  |  |
| up to 5.3 Euro | 1 |  |  |
| >5.3-10.6 Euro | 0.49 | 0.19- 1.30 | 0.152 |
| Greater than 10.6 Euro | 0.26 | 0.08- 0.91 | 0.020 |
| **Co-morbidity** |  |  |  |
| Yes | 1 |  |  |
| No | 2.44 | 0.67- 8.93 | 0.177 |
| **Anaesthesia type** |  |  |  |
| General | 1 |  |  |
| Loco-regional | 0.72 | 0.09- 5.63 | 0.757 |
| **Post-operative complications** |  |  |  |
| No | 1 |  |  |
| Yes | 0.52 | 0.11- 2.43 | 0.405 |
| **Duration of postoperative antibiotic** **therapy** |  |  |  |
| No post-operative antibiotic | 1 |  |  |
| 1-3 days | 1.68 | 0.47- 5.93 | 0.424 |
| More than 3 days | 1.41 | 0.37- 5.44 | 0.618 |
| **Post-operative length of stay** |  |  |  |
| Within 3 days | 1 |  |  |
| 4 days and longer | 0.52 | 0.29- 0.91 | 0.022 |
| **Total length of hospital stay** |  |  |  |
| Within 3 days | 1 |  |  |
| 4- 7 days | 0.71 | 0.41- 1.23 | 0.223 |
| **Counselling data collector** |  |  |  |
| Male data collectors | 1 |  |  |
| Female data collector | 2.26 | (1.25-12.83) | 0.019 |
| **Was the patient reminded of her appointment?** |  |  |  |
| No | 1 |  |  |
| Yes | 10.62 | 5.45- 20.71 | <0.001 |
| **Was it raining on the patient's appointment day?** |  |  |  |
| No | 1 |  |  |
| Yes | 0.74 | 0.28- 1.98 | 0.553 |
